# Plasma exchange in critically ill COVID-19 patients

**DOI:** 10.1186/s13054-020-03171-3

**Published:** 2020-08-04

**Authors:** Christian Morath, Markus A. Weigand, Martin Zeier, Claudius Speer, Shilpa Tiwari-Heckler, Uta Merle

**Affiliations:** 1 Department of Nephrology, Heidelberg University Hospital, Heidelberg, Germany; 2 Department of Anesthesiology, Heidelberg University Hospital, Heidelberg, Germany; 3 Department of Internal Medicine IV, Heidelberg University Hospital, Heidelberg, Germany

The spectrum of coronavirus disease 2019 (COVID-19) ranges from asymptomatic infection to respiratory failure and death of patients [1]. Severely affected patients may develop a cytokine storm-like clinical syndrome with multi-organ failure and a mortality rate of up to 90% [2]. Recently, it has been suggested that plasma exchange (PE) may positively influence this unfavorable course [3].

Here we report on five COVID-19 patients with a median age of 67 years who were admitted to the medical intensive care unit of Heidelberg University Hospital due to respiratory failure. Patients who had received at least one PE until May 15, 2020, were considered for analysis with clinical follow-up until June 15, 2020. Prophylactic antibiotic (piperacillin/tazobactam), antimycotic (caspofungin), and antiviral/immunomodulatory therapy (hydroxychloroquine or maraviroc) was initiated in all patients upon admission according to center practice. Additional treatments that were administered in some distance to PE are given in Table 1. During the course of the disease, patients developed vasopressor-dependent circulatory shock and/or persistent refractory fever (> 40.5 °C) together with increased interleukin 6 levels compatible with the cytokine storm-like clinical syndrome. In addition, all patients had multi-organ failure with acute respiratory distress syndrome (ARDS, 4 severe, 1 moderate) and acute kidney injury of at least KDIGO stage 2. A single PE with a median of 3.39 L of fresh frozen plasma was initiated in all patients followed by one additional treatment in patients 1, 3, and 5. During the PE, striking reduction of inflammatory markers C-reactive protein (− 47%, *P* = 0.0078) and interleukin 6 (− 74%, *P* = 0.0078), as well as significant reduction of ferritin (− 49%, *P* = 0.0078), LDH (− 41%, *P* = 0.0078), and D-dimer (− 47%, *P* = 0.016) were observed (Fig. 1a–e). Due to circulatory shock, four patients received vasopressor treatment at the start of the PE that could be substantially reduced during treatment (− 71%, *P* = 0.031, Fig. 1h). Biochemical and clinical improvement continued over the following days together with an increase in the oxygenation index in 4 out of 5 patients (Fig. 1i). These improvements were achieved with only 1 to 2 PE, which might be a possible indication of a direct pathophysiological influence of PE on the COVID-19-associated cytokine storm-like clinical syndrome. Three of the 5 most critically ill patients are alive, while a 71-year-old male and a 76-year-old female patient died after the therapy was limited due to persistent severe ARDS.

**Table 1:** Patient characteristics and treatment

	Patient 1	Patient 2	Patient 3	Patient 4	Patient 5
**Age (years)**	53	71	62	76	67
**Sex**	Male	Male	Male	Female	Female
**Comorbidities**	No	CAD, s/p CABG, schizophrenia, depression	Atrial fibrillation, hypertension	Diabetes, hypertension, s/p stroke	Diabetes, hypertension, CKD stage V^*^, obesity stage II
**Antibiotic therapy during the course of the disease**	Piperacillin/tazobactam^#^, azithromycin, meropenem, vancomycin, ceftazidime, metronidazole	Piperacillin/tazobactam^#^, azithromycin, meropenem, vancomycin	Piperacillin/tazobactam^#^, azithromycin, meropenem, vancomycin	Piperacillin/tazobactam^#^, meropenem, flucloxacillin	Piperacillin/tazobactam^#^
**Antifungal therapy during the course of the disease**	Caspofungin^#^	Caspofungin^#^	Caspofungin^#^	Caspofungin^#^	Caspofungin^#^
**Antiviral and immunomodulatory therapy during the course of the disease**	Hydroxychloroquine^#^, lopinavir/ritonavir, maraviroc^#^, aciclovir (for HSV)	Hydroxychloroquine^#^, maraviroc^#^, aciclovir (for HSV)	Maraviroc^#^, aciclovir (for HSV), ganciclovir (for CMV)	Maraviroc^#^	Maraviroc^#^, aciclovir (for HSV)
**Other therapy during the course of the disease**	Tocilizumab, interferon, prednisolone		Immunoglobulins, prednisolone, convalescent serum	Convalescent serum	
**Time from symptom to PE (days)**	12	9	16	17	11
**Time from admission to PE (days)**	6	4	8	10	5
**Processed plasma volume (L)**	3.60 3.66 (2)	3.38	3.02 2.93 (2)	3.17	3.51 3.40 (2)
**Clinical outcome as of June 15, 2020**	Extubated and spontaneous breathing, discharged from hospital	Died	Extubated and spontaneous breathing, discharged from hospital	Died	Extubated and spontaneous breathing, discharged from hospital

^*^Not yet on dialysis, ^#^representing center practice for critically ill COVID-19 patients at the time of treatment

**Fig. 1:**
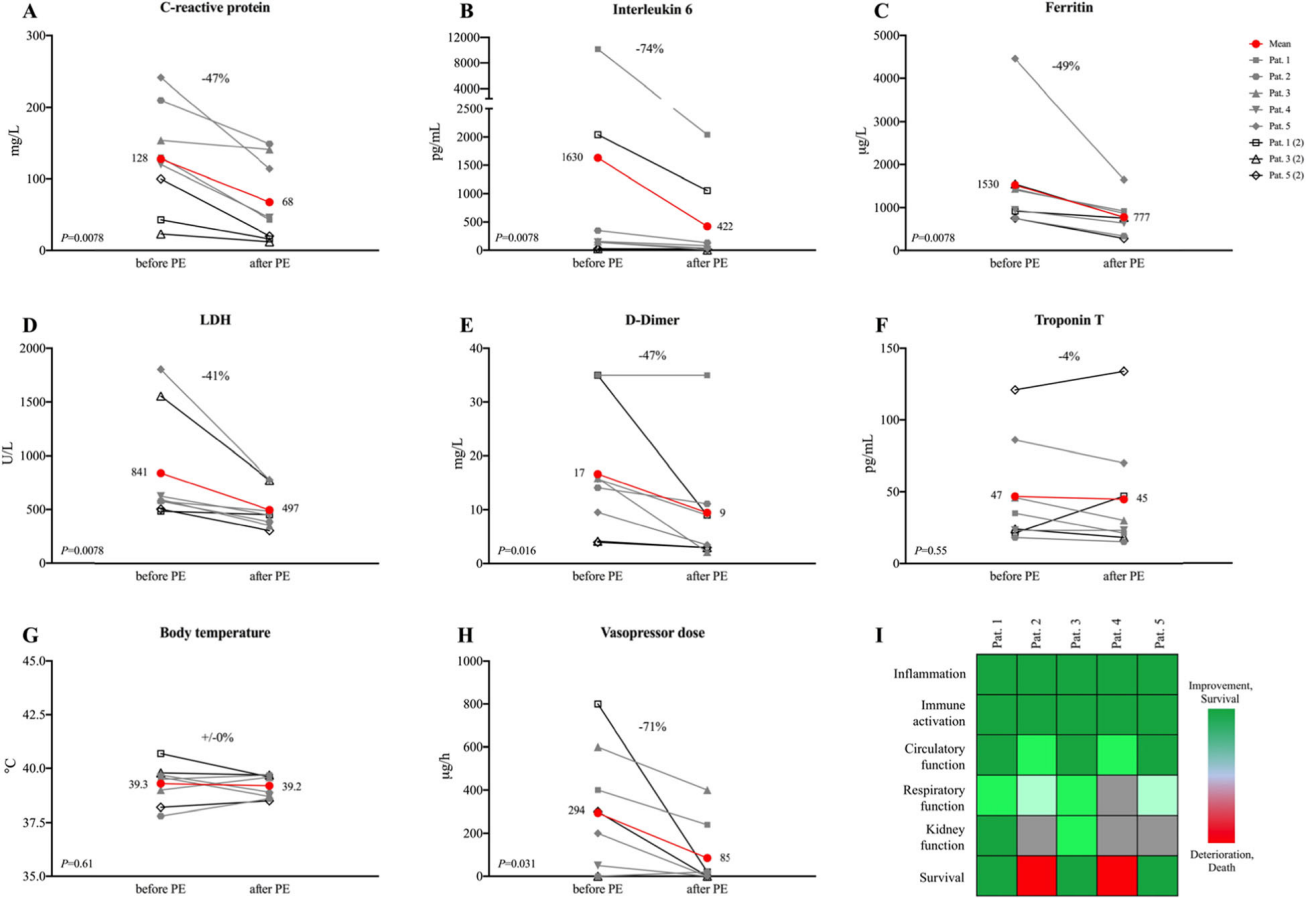
Changes of biochemical (**a**–**f**) and clinical (**g**–**i**) parameters during plasma exchange. **a**–**f** The first value after plasma exchange (PE) is compared to the last value before PE. **g**–**h** Mean values for the 24 h after compared to the 24 h before PE are given. Wilcoxon matched-pairs signed rank test was used for statistical analysis

It has been suggested that a cytokine storm-like clinical syndrome may be responsible for a significant proportion of COVID-19-associated patient deaths [4]. PE improved inflammation, microcirculatory clot formation, and hypotension, thereby improving clinical outcomes. Further studies to test whether (repeated) PE can alter the course of critically ill COVID-19 patients are clearly indicated.

## Data Availability

All data generated or analyzed during this study are included in this published article.
